# Canadian Psychiatry Human Resource Planning: Delphi-Method Study of Academic Chairs of Psychiatry of Canada: Planification des ressources humaines en psychiatrie au Canada : étude menée à l’aide des méthodes Delphi auprès des chefs de département de psychiatrie au Canada

**DOI:** 10.1177/07067437251408174

**Published:** 2026-01-16

**Authors:** Jitender Sareen, Corinne Isaak, Essence Perera, David A. Ross, Vincent Agyapong, Adekunle Garba Ahmed, Karin J. Neufeld, Gustavo Turecki, John Haggarty, Jessika Roy-Desruisseaux, Sarah Noble, Lakshmi N. Yatham, Patricia Hall, Simon Hatcher, Valerie Taylor, Pierre Gagnon, Zainab Samaan, Francois Lesperance, Benoit Mulsant

**Affiliations:** 1 Department of Psychiatry, University of Manitoba, Winnipeg, Manitoba, Canada; 2 Department of Psychiatry, University of Alberta, Edmonton, Alberta T6G 2H5, Canada; 3 Department of Psychiatry, Dalhousie University, Halifax, Nova Scotia B3H 4R2, Canada; 4 Department of Psychiatry, University of Saskatchewan, Saskatoon, Saskatchewan S7N 5E5, Canada; 5 Department of Psychiatry, McMaster University, Hamilton, Ontario L8P 1H6, Canada; 6 Department of Psychiatry, McGill University, Montreal, Quebec H3G 1A4, Canada; 7 Department of Psychiatry, Northern Ontario School of Medicine University, Sudbury, Ontario P3E 2C6, Canada; 8 Department of Psychiatry, Universite de Sherbrooke, Sherbrooke, Quebec J1H 5N4, Canada; 9 Department of Psychiatry, Memorial University, St. John's, Newfoundland and Labrador A1B 3V6, Canada; 10 Department of Psychiatry, University of British Columbia, Vancouver, British Columbia V6T 2A1, Canada; 11 Department of Psychiatry, Western University, London, Ontario N6A 5W9, Canada; 12 Department of Psychiatry, University of Ottawa, Ottawa, Ontario K1H 8M5, Canada; 13 Department of Psychiatry, University of Calgary, Calgary, Alberta T2N 4Z6, Canada; 14 Department of Psychiatry, Universite Laval, Québec, Quebec G1V 4G2, Canada; 15 Department of Psychiatry, Queen's University, Kingston, Ontario K7L 3N6, Canada; 16 Department of Psychiatry, University of Montréal, Montréal, Quebec H3T 1J4, Canada; 17 Department of Psychiatry, University of Toronto, Toronto, Ontario M5T 1P5, Canada

**Keywords:** psychiatry, workforce planning, health workforce, Canada, delivery of health care, mental health services

## Abstract

**Background:**

In 2023/2024, there were 15 psychiatrists/100,000 Canadians with inequitable distribution across Canada and unprecedented demand for mental health and addiction services. Psychiatry human resource planning in Canada has not occurred for more than a decade. The objectives of this study were to understand the current state and future directions related to Psychiatry Human Resources in the Canadian mental health care system.

**Methods:**

Using Delphi methods, we surveyed the 17 chairs of the academic departments of psychiatry in Canada and held focus groups. The Royal College and subspecialty programs were also engaged. Themes were extracted, summarized and refined. The refined themes were distributed via an online survey to all 17 chairs for final review and input, ensuring alignment and consensus across institutions.

**Results:**

Common themes focused on: the role of psychiatrists working in teams to provide care for complex mental disorders and addictions; need for innovative models of care including use of physician extenders, technology to reach the larger population of patients with mild to moderate disorders, working closely with primary care in collaborative care models. Due to the large proportion of Canadian psychiatrists being 35 years or more in practice (26%) and close to retirement, the chairs supported the need to expand the number of residency positions for psychiatry and continue strong recruitment efforts for international medical graduates. Although the majority of chairs supported shortening the general psychiatry residency program from 5 to 4 years, the Association of Chairs of Psychiatry of Canada (ACPC) could not reach a consensus on this issue. Pan-Canadian licensing for psychiatrists should be considered due to inequitable distribution of psychiatrists in Canada and advances in virtual care post-COVID-19 pandemic.

**Conclusions:**

This study will contribute to the dialogue on psychiatry human resources planning in Canada.

## Plain Language Summary Title

Psychiatrist Human Resource Planning in Canada according to Chairs of 17 Canadian Psychiatry Departments

Plain Language Summary

Canada is facing a shortage and uneven distribution of psychiatrists. This study asked psychiatry leaders how to improve care. Key suggestions included using technology, training more doctors, working in teams, and making licensing easier across provinces. Their ideas aim to better meet growing mental health and addiction care needs.

## Graphical abstract

**Graphical abstract:**
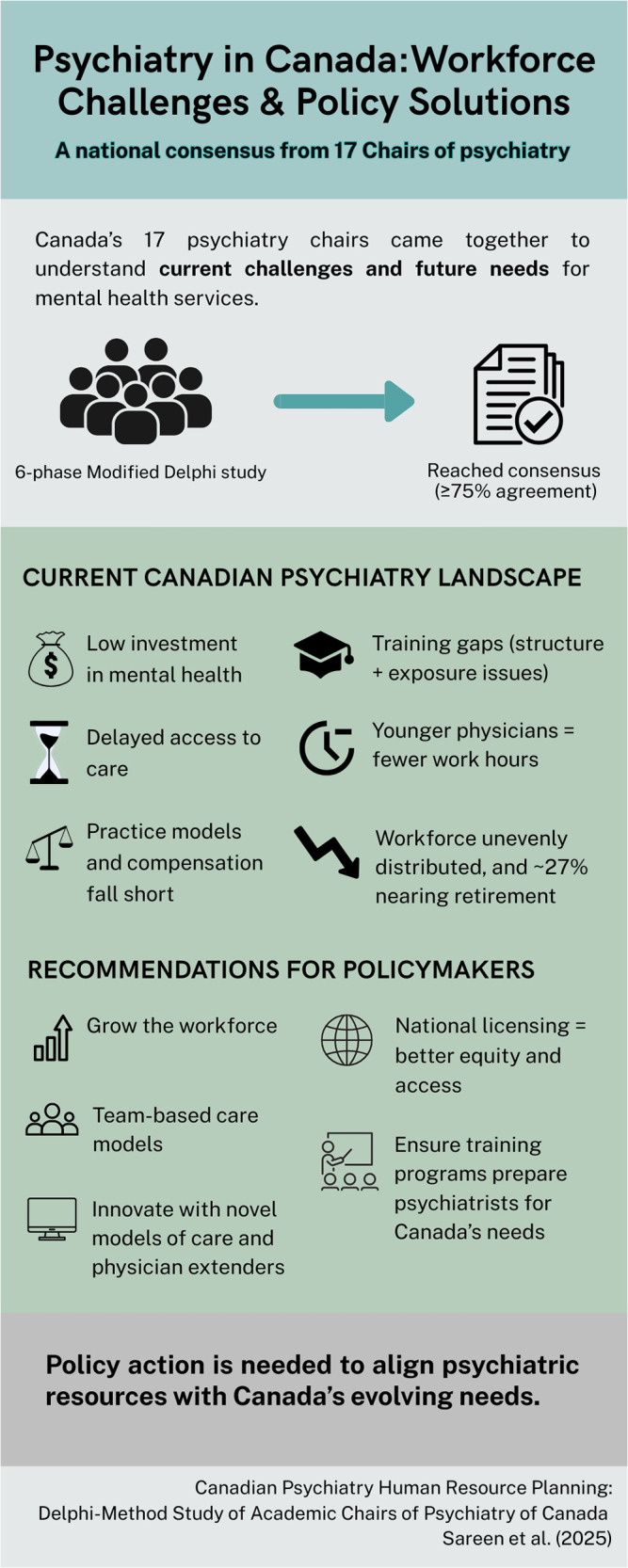
This is a visual representation of the abstract.

## Résumé

**Contexte:**

En 2023–2024, il y avait 15 psychiatres pour 100 000 Canadiens répartis de façon inéquitable au Canada, et la demande de services de santé mentale et de traitement des dépendances était sans précédent. Les ressources humaines en psychiatrie au Canada n’ont fait l’objet d’aucune planification depuis plus d’une décennie. Cette étude visait à comprendre la situation actuelle et les orientations futures des ressources humaines en psychiatrie dans le système de soins en santé mentale du Canada.

**Méthodes:**

À l’aide des méthodes Delphi, nous avons sondé les 17 chefs de département de psychiatrie au Canada et tenu des groupes de discussion. Les programmes du Collège royal et des sous-spécialités ont également été mis à contribution. Les thèmes ont été extraits, résumés et cernés. Les thèmes cernés ont fait l’objet d’un sondage en ligne auprès des 17 chefs pour un examen final et la sollicitation de commentaires pour assurer l’harmonisation et le consensus entre les établissements.

**Résultats:**

Les thèmes communs portaient sur le rôle des psychiatres qui travaillent en équipe pour fournir des soins dans le contexte de troubles mentaux complexes et de dépendances; le besoin de modèles de soins novateurs, y compris le recours à des adjoints, la technologie pour atteindre une population élargie de patients atteints de troubles légers à modérés; une collaboration étroite avec les milieux de soins primaires dans des modèles de soins axés sur la collaboration. Compte tenu de la forte proportion de psychiatres canadiens qui cumulent au moins 35 ans (26%) et qui sont sur le point de prendre leur retraite, les chefs appuient la nécessité d’accroître le nombre de postes de résidence en psychiatrie et de poursuivre les grands efforts de recrutement de diplômés internationaux en médecine. Bien que la majorité des chefs appuient la réduction de cinq à quatre ans de la durée du programme de résidence en psychiatrie générale, l’Association des chefs de département de psychiatrie du Canada (ACDPC) n’a pu parvenir à un consensus sur cette question. Il faudrait envisager d’accorder aux psychiatres un permis pancanadien en raison de leur répartition inéquitable au Canada et des progrès réalisés dans les soins virtuels après la pandémie de COVID-19.

**Conclusions:**

Cette étude contribuera au dialogue sur la planification des ressources humaines en psychiatrie au Canada.

## Introduction

A 2024 Canadian Mental Health Association State of Mental Health in Canada reports six key national challenges to improving mental health and addiction care: worsening mental health in the population, underfunding of mental health and addiction services both federally and provincially, geographic disparities (especially for rural and indigenous communities), insufficient data, and the need for legislative reform.^1^ There is unprecedented demand for mental health and addiction services in Canada. Many jurisdictions have long wait times for accessing mental health and addiction care. Canadian Psychiatric Association (CPA) benchmarks are that elective assessments should occur within 1 month of the consultation from primary care providers.^2^ However, most jurisdictions in Canada currently have waiting times well beyond 3 months, with some over a year.^3^ Psychiatrists are an important part of the mental health care system, and Psychiatry Human Resource (PHR) planning in Canada has not occurred for more than a decade.

### How Many Psychiatrists are Required in a Population?

The World Health Organization (WHO) recommends a psychiatrist-to-population ratio of 1:10000. This figure originated in 1962 and was later adopted by the American Psychiatric Association. Since then both Canada and the United States (US) have found the ratios to be inadequate, and many developed countries have advocated for a range between one psychiatrist for every 6,500 to 10,000.^4^ The 2010 CPA PHR Position paper recommended a clinical psychiatric full-time equivalent (FTE) to population ratio of 1:8400 and a licensed psychiatrist to population ratio of 1:6584.^2^ The CPA paper quotes 2005 Canadian Institutes of Health Information (CIHI) data demonstrating that there were 4,140 psychiatrists in Canada with a ratio of 1:8925. Yet, Kurdyak et al. have shown that in Ontario, increasing the number of psychiatrists is not necessarily associated with an increase in access to psychiatry consultations and treatment.^5–7^ Using data from Ontario, Kurdyak et al. (2014) examined practice patterns of full-time psychiatrists (1,379) and found that in high supply areas, 40% psychiatrists saw less than 100 new patients per year.^7^ These patients were more frequently seen, had a lower likelihood of previous hospitalizations, and had higher socioeconomic status. Kurdyak et al. (2014) note that “increasing the supply of psychiatrists while funding unlimited frequency and duration of psychotherapy care may not improve access for patients who need psychiatric services.”^7^ Recent findings from Rudoler et al. (2025) reinforce this concern, highlighting that the number of psychiatrists per capita remained stable between 2012 and 2022 across British Columbia, Manitoba and Ontario, despite rising demand.^8^ Supply was highest in large urban centres, yet only moderately correlated with ongoing care and not correlated with brief consultations, suggesting that increasing psychiatrist numbers alone is insufficient to address access issues.^8^

The CPA PHR paper^4^ delineates the complexities of the estimation of need per population and the limitation of such a population ratio and recommends an activity-based assessment of psychiatrists rather than the number of licensed psychiatry ratio. Faulkner and Goodman^9^ recommend estimating PHR requirements based on patients’ needs: 1) psychiatric FTEs, and the proportion of psychiatry FTE available for direct patient care, 2) demands for psychiatry based on specific system needs, 3) estimates based on data from other countries, 4) estimates based on how many psychiatrists the system can afford, and 5) number of patients that need psychiatric care. The CPA is revising its Health Human Resource (HHR) policy paper currently.

The Association of Chairs of Psychiatry of Canada (ACPC) has representation from all 17 psychiatry departments from across Canada. The chairs of psychiatry are responsible for academic missions (research, undergraduate, and postgraduate missions) of the department. Many chairs also have a health system leadership role that includes oversight of clinical psychiatric services. HHR planning for the individual departments and often health systems in their region are a major role and responsibility of the chairs. To date, there has never been a collective national statement from the chairs of psychiatry on HHR planning in Canada.

This paper uses a Delphi Method approach to create a consensus statement of the ACPC chairs on HHR psychiatry planning in Canada. We aimed to: 1) document the current state of PHR in Canada, and 2) recommend future directions related to psychiatry staffing.

## Methods

The Delphi Method or Delphi Technique is a common research method used to reach consensus about a specific issue or complex problem when there is limited evidence or contentiousness.^10^ It is a structured yet iterative process that allows for amalgamation of information^11^ related to the issue and decision-making by a group of experts^12^ in a particular field. Experts typically go through several rounds of consensus making to come to an agreement.^13^ A modified Delphi method was used to develop a national consensus on PHR planning by the ACPC.^11^ The modified Delphi method is a structured approach to expert consensus that uses guided discussions and iterative surveys to efficiently refine and prioritize solutions, making it well suited for multifaceted issues.^11^

There were six phases for the study. Readers interested in the details of the process and surveys utilized in this study can contact the corresponding author.

Phase 1: Identification of Need. In Spring 2024, ACPC members agreed that a national position on PHR planning was necessary.

Phase 2: Pre-Meeting Survey. In October 2024, a survey was distributed electronically to all 17 departmental chairs via SurveyMonkey data (SurveyMonkey Inc., San Mateo, California, USA). The survey collected qualitative and quantitative data on the current state of psychiatry in each department and province, as well as suggestions for future directions at departmental and system levels.

Phase 3: In-Person Meeting. In November 2024, ACPC held an in-person meeting to review survey findings and identify preliminary themes. All 17 chairs were present (virtually or in person). Representatives from the CPA, Royal College of Physicians and Surgeons of Canada, Canadian Academy of Psychiatric Leadership (CAPL), Canadian Academy of Child and Adolescent Psychiatry (CACAP), and Canadian Academy of Geriatric Psychiatry (CAGP) provided invited presentations to inform the discussion.

Phase 4: Thematic Analysis. Between December 2024 and March 2025, a research associate (CI) conducted a qualitative thematic analysis of the open-ended survey responses using SurveyMonkey data (SurveyMonkey Inc., San Mateo, California, USA). Key themes were synthesized and categorized.

Phase 5: Consensus Refinement. In March 2025, the majority of ACPC members (10/17) met to review and refine the emerging themes.

Phase 6: Final Review. In May 2025, the revised themes were circulated electronically via online survey through SurveyMonkey to all 17 chairs for final review and feedback, ensuring consensus across institutions (SurveyMonkey Inc., San Mateo, California, USA).

Consensus was predefined with 75% or more of the ACPC members (13/17) endorsing the statement. Complete consensus was defined as 100% endorsement of the statement (17/17).

Statements were grouped into three categories, ACPC consensus statements on the current state in Canada (nine statements), ACPC recommendations for policymakers on PHR planning (five statements) and ACPC issues discussed that were not included in the consensus statements (two statements). Experts were able to indicate whether they endorse the statement as written, do not endorse the statement, or recommend removing the statement entirely. Experts who did not endorse or recommended removing a specific statement were invited to propose revisions.

This structured, iterative process supported the development of a pan-Canadian consensus on priorities and strategies for psychiatry workforce planning. This project was conducted as a quality improvement initiative and, as such, did not require review by the institutional research ethics board.

## Results

Table 1 shows the latest available 2023 CIHI data on the number of psychiatrists in Canada.^14^ In 2023, there were 5,819 psychiatrists (i.e., 15 psychiatrists/100,000 or 1 psychiatrist for 6,670). In 2023, 27% of the psychiatrists practicing in Canada were 36 or more years in practice, and 67% were Canadian Medical graduates. There were less than five psychiatrists registered in the Territories as their primary license. Much of the service in Nunavut, and Yukon is provided by psychiatrists registered in other provinces through itinerant and/or telehealth. Among provinces, Saskatchewan and Prince Edward Island had the lowest prevalence of psychiatrists (9/100,000 and the highest in Nova Scotia (19/100,000) and British Columbia (17/100,000).

**Table 1.** 2023 Canadian Institute of Health Information (CIHI) Data on Psychiatrists in Canada.

	NL	PEI	NS	NB	QC	ON	MB	SK	ALTA	BC	Canada
Number of psychiatrists	83	15	200	101	1,326	2,195	190	108	645	948	5,816
Psychiatrists/100,000	15	9	19	12	15	14	13	9	14	17	15
Number of Canadian MD graduate	55	9	114	41	1,077	1,425	142	48	408	590	3,912
Years in practice	< 5	6–10	11–15	16–20	21–25	26–30	31–35	> 36	Unknown	Total	
Number of psychiatrists	67	761	738	673	712	668	511	1,556	133	5,819	
Percent of total	1.2%	13.1%	12.7%	11.6%	12.2%	11.5%	8.8%	26.7%	2.3%		

*Note.* This table is derived from CIHI data.^14^ The count for Canada includes psychiatry care provided by physicians residing in the Territories. Itinerant psychiatry care provided in the Territories is not captured, only those living in the communities in 2023. ALTA = Alberta; BC = British Columbia; MB = Manitoba; NB = New Brunswick; NL = Newfoundland and Labrador; NWT = Northwest Territories; NS = Nova Scotia; ON = Ontario; PEI = Prince Edward Island; QC = Quebec; SK = Saskatchewan.

Table 2 displays the current psychiatry faculty and number of residents being trained in Canadian academic departments as of May 2025. As of May 2025, there were a total of 4,027 psychiatry faculty members (full-time and part-time) and 1,119 psychiatry residents across Canadian departments of psychiatry. On average, 284 general psychiatry residency spots were available annually nationwide. Subspecialty training programs in child and adolescent psychiatry, geriatric psychiatry, and forensic psychiatry showed significantly lower capacity, with annual averages of 52, 34, and 13 training positions, respectively. The University of Toronto and the University of British Columbia had the highest faculty counts, each with 700 psychiatry faculty members. Correspondingly, they also supported the largest psychiatry residency cohorts, with 200 and 140 residents, respectively. University of Toronto offered the most general residency spots per year (40), followed by UBC (30), and Memorial University (30).

**Table 2.** Psychiatry Faculty and Residency Training Programs Across Canadian Psychiatry Departments as of May 2025.

	Psychiatry faculty (Full-time and part-time)	Psychiatry residents	Child and adolescent residents	Geriatric residents	Forensic residents
	Total number	Total number (Average spots/year)	Total number (Average spots/year)	Total number (Average spots/year)	Total number (Average spots/year)
Memorial University	60	28 (30)	1 (1)	1 (0)	0 (0)
Université de Sherbrooke	61	63 (9)	2 (2)	0 (0)	0 (0)
Queen's University	70	38 (38)	2 (2)	2 (2)	0 (0)
Western University	95	65 (13)	2 (2)	0 (2)	0 (0)
Dalhousie University	103	41 (9)	2 (2)	1 (1)	0 (0)
Northern Ontario School of Medicine University	104	22 (6)	0 (0)	0 (0)	0 (0)
University of Saskatchewan	125	34 (7)	4 (3)	0 (0)	2 (1)
Université Laval	140	65 (13)	2 (4)	2 (4)	0 (0)
University of Manitoba	161	59 (15)	5 (3)	2 (1)	0 (0)
University of Alberta	200	50 (10)	6 (3)	4 (4)	0 (2)
McGill University	249	61 (10)	1 (5)	2 (4)	0 (0)
University of Ottawa	250	65 (16)	1 (2)	2 (1)	1 (2)
McMaster University	296	47 (11)	5 (2)	2 (1)	1 (2)
University of Calgary	350	53 (10)	2 (5)	1 (2)	0 (1)
Université de Montréal	363	88 (17)	2 (4)	2 (4)	1 (2)
University of Toronto	700	200 (40)	8 (4)	6 (4)	0 (2)
University of British Columbia	700	140 (30)	16 (8)	2 (4)	2 (1)
Total in Canada	4,027	1,119 (284)	61 (52)	29 (34)	7 (13)

Subspecialty distribution varied widely by institution. Child and adolescent psychiatry programs were present in most institutions, with University of British Columbia hosting the largest cohort (16 total, averaging eight per year). Forensic psychiatry training was the least common, with only seven programs nationwide. Notably, only Université de Montréal, University of Ottawa, McMaster University, and University of British Columbia reported active forensic psychiatry training, each offering an average of 1–2 spots per year. Geriatric psychiatry programs were more widespread, but also limited in size, with an annual national average of only 34 training positions. Several institutions, including Northern Ontario School of Medicine, Université de Sherbrooke, and Memorial University, did not offer any subspecialty training in forensic or geriatric psychiatry, and had minimal child and adolescent psychiatry capacity. Overall, the data shows that there are large disparities in the faculty size and training programs across the country, particularly in subspecialty areas.

Table 3 presents the summary of the 16 statements which reached consensus from this group of experts. Survey responses were received from all 17 experts. All statements reached consensus, with over 75% agreement among the chairs. Experts who did not endorse or recommended removing a specific statement were invited to propose revisions, see appendix for these revised statements. Complete consensus (i.e., 100% agreement) was achieved by the group of 17 experts on eight key statements. These statements highlighted persistent delays in accessing elective psychiatric assessments across most jurisdictions, inequitable distribution of psychiatrists despite increases in their overall numbers, and the aging of the psychiatric workforce, with approximately 27% nearing retirement age. The group also agreed that increasing the number of psychiatrists alone does not necessarily improve access to care, and that younger physicians are tending toward reduced workloads compared to older cohorts. Concerns were raised about psychiatry training in Canada, including its length, patient volumes during residency, and fragmentation of core training. The experts agreed that psychiatry training programs must ensure graduates are equipped to meet Canada's population needs in clinical care, education, and research. Finally, consensus was reached that defining the specific population needs for psychiatric subspecialists is beyond the scope of the current work and should be undertaken by subspecialty associations.

**Table 3.** Summary of Statements from the Delphi Method.

	Responses from experts (total of 17 chairs)		
	I endorse this statement as written.	I do not endorse this statement.	I recommend removing this statement entirely.
ACPC consensus statements on the current state in Canada	N/Total (%)	N/Total (%)	N/Total (%)
1. In comparison with other developed countries, Canada invests less funding for mental health and substance use services.	16/17 (94.1%)	0/17 (0.0%)	1/17 (5.9%)
2. Canada has seen an increase in the prevalence of mental disorders and substance use disorders over the past 10 years.	16/17 (94.1%)	1/17 (5.9%)	0/17 (0.0%)
3. Most jurisdictions have delays in accessing elective psychiatric assessment well beyond the Canadian Psychiatric Association benchmark of 30 days.	17/17 (100.0%)	0/17 (0.0%)	0/17 (0.0%)
ACPC consensus statements on the current state in Canada			
4. Although Canada has had an increase in the number of psychiatrists per population (2010: 4,140 psychiatrists with a ratio of 1:8925; 2023: 5,819 with a ratio of 1:6670), psychiatrists are inequitably distributed in urban centres and there are disparities across provinces and territories.	17/17 (100.0%)	0/17 (0.0%)	0/17 (0.0%)
5. According to 2023 Canadian Institute of Health Information data, approximately 27% of Psychiatrists are nearing retirement age (36 + years in practice as a psychiatrist).	17/17 (100.0%)	0/17 (0.0%)	0/17 (0.0%)
ACPC consensus statements on the current state in Canada			
6. The current models of psychiatric practice and compensation are not working in adequately meeting the mental health care needs of the population.	15/17 (88.2%)	2/17 (11.8%)	0/17 (0.0%)
7. Data from some jurisdictions in Canada suggest that simply increasing the number of psychiatrists does NOT increase access to psychiatric care.	17/17 (100.0%)	0/17 (0.0%)	0/17 (0.0%)
8. There is a trend among younger physicians towards decreasing workloads compared to older cohorts of physicians.	17/17 (100.0%)	0/17 (0.0%)	0/17 (0.0%)
ACPC consensus statements on the current state in Canada			
9. There were concerns related to the psychiatry training in Canada (e.g., length of training, volumes of patients seen during residency, and fragmentation of core training).	17/17 (100.0%)	0/17 (0.0%)	0/17 (0.0%)
**ACPC consensus recommendations for policymakers on PHR planning**	**N/Total (%)**	**N/Total (%)**	**N/Total (%)**
10. There is a need for training and recruiting more psychiatrists in Canada to keep up with the current ratio of psychiatrists in the Canadian population because of the aging workforce, and younger cohorts working less than older cohorts.	15/17 (88.2%)	2/17 (11.8%)	0/17 (0.0%)
ACPC consensus recommendations for policymakers on PHR planning			
11. Psychiatric practice models should occur primarily within multidisciplinary teams and primary care settings to expand the capacity of psychiatry to provide care to more people with mental disorders.	16/17 (94.1%)	1/17 (5.9%)	0/17 (0.0%)
12. There is a need for novel models of care (e.g., eConsult) and the use of physician extenders (e.g., physician assistants and clinical assistants) to expand the reach of psychiatry in the population.	15/17 (88.2%)	1/17 (5.9%)	1/17 (5.9%)
ACPC consensus recommendations for policymakers on PHR planning			
13. Pan-Canadian licensing for psychiatrists should be considered due to inequitable distribution of psychiatrists in Canada and advances in virtual care post COVID-19 pandemic.	15/17 (88.2%)	0/17 (0.0%)	2/17 (11.8%)
14. Psychiatry training programs should ensure that newly trained psychiatrists can respond to the population needs in Canada from a clinical, education, and research perspective.	17/17 (100.0%)	0/17 (0.0%)	0/17 (0.0%)
ACPC issues discussed that were not included in the consensus statements			
15. Although the majority of chairs supported the idea of reducing the length of general psychiatry training to 4 years from the current 5 years (similar to US training, and some other specialties), ACPC could not reach a consensus on this issue. Further work is needed to explore the similarities and differences between Canadian and US training and whether some Canadian schools may want to pilot an accelerated 4-year psychiatry residency program similar to the US.	15/17 (88.2%)	1/17 (5.9%)	1/17 (5.9%)
ACPC issues discussed that were not included in the consensus statements			
16. ACPC was not able to reach a consensus on the specific population needs of subspecialists in Canada. Although input from subspecialty associations was garnered, ACPC members felt that it was beyond the scope of the current paper and that this work needed to be done by the subspecialty associations.	17/17 (100.0%)	0/17 (0.0%)	0/17 (0.0%)

*Note.* N = Number of experts.

PHR, Psychiatry Human Resource; US, United States.

## Discussion

To the best our knowledge, this is the first consensus statement on HHR planning by chairs of psychiatry in any country. With the backdrop of an underfunded and fragmented Canadian health care system, and long waiting times for psychiatric assessments and treatment,^1^ aging psychiatric workforce, trends among all Canadian physicians for decreasing work hours,^15^ there was support for increasing the number of trainees in residency programs and recruiting psychiatrists to even keep up with the current psychiatry population rates. There was strong consensus on considering novel models of care, use of physician extenders, to ensure equitable access to psychiatric care. Due to the advances in virtual care, unequitable distribution of psychiatrists across Canada,^8^ recommendations were to consider pan-Canadian licensing for psychiatrists. Finally, there was strong support for training programs to ensure that training is aligned with psychiatrists being able to have a strong population impact on psychiatric services.

The current study builds on the work done by CPA's Position paper in 2010 and contributes to the upcoming revision of the CPA Position paper. Although the number of psychiatrists in Canada has grown over the last 15 years, due to lack of data on the number of patients seen by the psychiatrists, it remains unknown whether the gaps between need and access have increased or decreased in Canada. Anecdotal data suggests, especially post-COVID-19 pandemic, that there is a greater gap between need and access. We speculate that this increase in gap is related to the substantial rise in mental health problems in the Canadian population and reductions in stigma over the decades. Further, our results demonstrated that there are substantial disparities in psychiatry faculty and training capacity across Canadian academic institutions as of May 2025. While a few large, urban universities maintain well-resourced departments with extensive faculty and residency programs, many smaller or regionally located institutions operate with fewer faculty members and trainees. This uneven academic infrastructure mirrors broader trends in the distribution of the psychiatry workforce and likely contributes to persistent regional disparities in access to mental health services. These findings align with those of Rudoler et al. (2025), who documented significant geographic variation in psychiatrist supply and service utilization across British Columbia, Manitoba, and Ontario.^8^

There were no studies internationally that we could compare our findings with. However, most international recommendations focus on a team-based model for psychiatrists to reach as many patients as possible with a mental disorder. In 2025, CIHI has published a report with respect to HHR planning for all physicians in Canada over the next 20 years based on increases in medical school and residency sizes by 10%.^16^ Specific HHR projections for psychiatry residency expansions and impact on workforce in Canada are required.

## Strengths and Limitations

A major strength of the process was the structured, iterative design, which included both qualitative and quantitative data collection, expert stakeholder engagement, and multiple rounds of feedback. The inclusion of representatives from national psychiatric organizations (CPA, Royal College, CAPL, CACAP, CAGP) added breadth and relevance to the discussions. Consensus was defined a priori as 75% or more agreement among respondents for each proposed statement. This clear threshold ensured that the final statements reflected strong collective support. However, some limitations must be acknowledged. While broad in representation, the process was limited to department chairs and may not fully capture perspectives from frontline clinicians, early-career psychiatrists, or trainees. Additionally, while numerous topics were discussed, only those that met the consensus threshold or were deemed within the ACPC's mandate were included in the final statement. Some important but unresolved or jurisdiction-specific issues were excluded due to either lack of consensus or agreement that they fell outside the scope of the ACPC's role. Another limitation is that our group did not model the impact of the number of current psychiatry residents and impact on the future supply of psychiatrists in Canada. Future studies could explore this important modelling to guide the expansion of residency programs and recruitment of psychiatrists. Lastly, as with any consensus-based methodology, there is a risk that minority viewpoints may be underrepresented. Nonetheless, this process represents a rigorous and transparent effort to articulate national priorities in psychiatric workforce planning.

Future research directions on PHR planning in Canada include the following:

1. Detailed comparison of US and Canadian residency programs with the potential for creating an accelerated 4-year Canadian psychiatry training program. This work should include engagement of residents, medical students (whether a shorter residency would garner more interest in psychiatry), and key stakeholders.
2. The use of similar Delphi Methods to develop consensus statements on PHR for subspecialties in psychiatry.
3. Systematic Engagement of patient and families’ perspectives in PHR planning in Canada.
4. More controlled evaluations are required on the utility and impact of physician extenders (physician assistants, clinical assistant, human scribes and artificial intelligence scribes) to increase the efficiency and reduce the administrative burden on psychiatrists.^17^
5. Further research could also examine the role of international medical graduates in Canada. CIHI reports that 23% of Canadian psychiatrists are International medical graduates.^14^ PHR planning should consider expansion of psychiatry residency spots in Canada as well as a specific plan for recruitment of International medical graduates.
6. Novel models of care including e-consults, group medical visits,^18^ rapid access to consultative expertise,^19^ virtual hallway have been variably implemented across different Canadian provinces.^20^ Evidence-based, cost-effective models of care should be spread across Canadian provinces in a systematic manner with careful evaluations.
7. Working with Federal and Provincial regulatory bodies to delineate the potential impacts of pan-Canadian licensing of psychiatrists.

## Conclusions

The current paper provides consensus statements of the current state and recommendations from the academic chairs of psychiatry departments using a structured Delphi approach to HHR planning in Canada. This paper contributes to the larger dialogue with partners across the Federal, provincial governments, Royal College of Physicians and Surgeons, CPA, and subspecialties on improving the mental health system for Canadians.

## Supplemental Material

sj-docx-1-cpa-10.1177_07067437251408174 - Supplemental material for Canadian Psychiatry Human Resource Planning: Delphi-Method Study of Academic Chairs of Psychiatry of Canada: Planification des ressources humaines en psychiatrie au Canada : étude menée à l’aide des méthodes Delphi auprès des chefs de département de psychiatrie au Canada Supplemental material, sj-docx-1-cpa-10.1177_07067437251408174 for Canadian Psychiatry Human Resource Planning: Delphi-Method Study of Academic Chairs of Psychiatry of Canada: Planification des ressources humaines en psychiatrie au Canada : étude menée à l’aide des méthodes Delphi auprès des chefs de département de psychiatrie au Canada by Jitender Sareen, Corinne Isaak, Essence Perera, David A. Ross, Vincent Agyapong, Adekunle Garba Ahmed, Karin J. Neufeld, Gustavo Turecki, John Haggarty, Jessika Roy-Desruisseaux, Sarah Noble, Lakshmi N. Yatham, Patricia Hall, Simon Hatcher, Valerie Taylor, Pierre Gagnon, Zainab Samaan, Francois Lesperance and Benoit Mulsant in The Canadian Journal of Psychiatry

## Appendix

**Appendix:** Revised statements from experts who did not endorse or recommended removal of each statement.

ACPC consensus statements on the current state in Canada	Revised statement
1.In comparison with other developed countries, Canada invests less funding for mental health and substance use services.	‘I suggest any reference point should be what WHO recommends as an acceptable level of funding for mental health and substance use services relative to general health care’
2.Canada has seen an increase in the prevalence of mental disorders and substance use disorders over the past 10 years.	'Canada has seen an increase in the number of persons looking for services for mental disorders and substance use disorders over the past 10 years.'
6. The current models of psychiatric practice and compensation are not working in adequately meeting the mental health care needs of the population.	‘The existing models of psychiatric practice and compensation fail to adequately meet the mental health care needs of the population, necessitating a restructured approach that actively incorporates academic engagement and equitable access to resources, regardless of geographical location.’ ‘In some areas of Canada… the current model… I believe in some areas; psychiatrists are paid well.’
ACPC consensus recommendations	Revised statement
10. There is a need for training and recruiting more psychiatrists in Canada to keep up with the current ratio of psychiatrists in the Canadian population because of the aging workforce, and younger cohorts working less than older cohorts.	‘I believe in many areas of Canada; there are enough psychiatrists. but they are poorly distributed.’ ‘There is a need for changing the way psychiatrists practice in Canada because of the aging workforce, and younger cohorts working less than older cohorts.’
11. Psychiatric practice models should occur primarily within multidisciplinary teams and primary care settings to expand the capacity of psychiatry to provide care to more people with mental disorders.	‘Psychiatric practice models should be grounded in multidisciplinary team-based care and integrated across all levels of the healthcare system — from primary to tertiary — to ensure broad access, enhance quality of care, and advance meaningful research in psychiatry.’
12. There is a need for novel models of care (e.g. eConsult) and the use of physician extenders (e.g. physician assistants) to expand the reach of psychiatry in the population.	‘I agree with eConsult but I see no reason the use of physician extenders.’ ‘eConsults do not reduce the need for a number of psychiatrists, as the time spent is similar whether it is in person or by eConsult. As to physician extenders, I am not convinced that they will add to the capacity without compromising the quality of care’
13. Pan-Canadian licensing for psychiatrists should be considered due to inequitable distribution of psychiatrists in Canada and advances in virtual care post COVID-19 pandemic.	‘I am not aware of evidence that supports open licensure as a way to improve access. Rather, it may worsen it due to funding differences.’ ‘Most psychiatric care required informed knowledge of local community resources and the need to work in teams with other local professionals. Fragmenting the number of psychiatrists working in remote areas thru virtual care will not improve the accountability and the quality of team-based practice.’
Issues discussed that were not included in the consensus statements	Revised statement
15.Although the majority of chairs supported the idea of reducing the length of general psychiatry training to 4 years from the current 5 years (similar to US training, and some other specialties), ACPC could not reach a consensus on this issue. Further work is needed to explore the similarities and differences between Canadian and US training and whether some Canadian schools may want to pilot an accelerated 4-year psychiatry residency program similar to the US.	‘I disagree with the reducing the duration of training, as even with a 5-year training, many residents do not feel competent to practice independently. If reduction in training is a consideration, then we need to look at increasing their caseloads to ensure they get adequate experience and training’ ‘I agree but did not realize the “majority” requests this. I thought it was otherwise.’

WHO, World Health Organization; US, United States.
