# Supplementary material for: Canadian Psychiatry Human Resource Planning: Delphi-Method Study of Academic Chairs of Psychiatry of Canada: Planification des ressources humaines en psychiatrie au Canada : étude menée à l’aide des méthodes Delphi auprès des chefs de département de psychiatrie au Canada
Source: Can J Psychiatry. 2026 Jan 16;71(3):174–84. doi: 10.1177/07067437251408174 (PMC12811089; doi:10.1177/07067437251408174)
Supplement: sj-docx-1-cpa-10.1177_07067437251408174 - Supplemental material for Canadian Psychiatry Human Resource Planning: Delphi-Method Study of Academic Chairs of Psychiatry of Canada: Planification des ressources humaines en psychiatrie au Canada : étude menée à l’aide des méthodes Delphi auprès des chefs  [file sj-docx-1-cpa-10.1177_07067437251408174.docx]

**Appendix: Revised statements from experts who did not endorse or recommended removal of each statement.**

| **ACPC Consensus Statements on the current state in Canada** | **Revised Statement** |
| --- | --- |
| 1.In comparison with other developed countries, Canada invests less funding for mental health and substance use services. | ‘I suggest any reference point should be what WHO recommends as an acceptable level of funding for mental health and substance use services relative to general health care’ |
| 2.Canada has seen an increase in the prevalence of mental disorders and substance use disorders over the past 10 years. | 'Canada has seen an increase in the number of persons looking for services for mental disorders and substance use disorders over the past 10 years.' |
| 6. The current models of psychiatric practice and compensation are not working in adequately meeting the mental health care needs of the population. | ‘The existing models of psychiatric practice and compensation fail to adequately meet the mental health care needs of the population, necessitating a restructured approach that actively incorporates academic engagement and equitable access to resources, regardless of geographical location.’  ‘In some areas of Canada... the current model... I believe in some areas; psychiatrists are paid well.’ |
| **ACPC Consensus Recommendations** | **Revised Statement** |
| 10. There is a need for training and recruiting more psychiatrists in Canada to keep up with the current ratio of psychiatrists in the Canadian population because of the aging workforce, and younger cohorts working less than older cohorts. | ‘I believe in many areas of Canada; there are enough psychiatrists. but they are poorly distributed.’  ‘There is a need for changing the way psychiatrists practice in Canada because of the aging workforce, and younger cohorts working less than older cohorts.’ |
| 11. Psychiatric practice models should occur primarily within multidisciplinary teams and primary care settings to expand the capacity of psychiatry to provide care to more people with mental disorders. | ‘Psychiatric practice models should be grounded in multidisciplinary team-based care and integrated across all levels of the healthcare system — from primary to tertiary — to ensure broad access, enhance quality of care, and advance meaningful research in psychiatry.’ |
| 12. There is a need for novel models of care (e.g. eConsult) and the use of physician extenders (e.g. physician assistants) to expand the reach of psychiatry in the population. | ‘I agree with eConsult but I see no reason the use of physician extenders.’  ‘eConsults do not reduce the need for a number of psychiatrists, as the time spent is similar whether it is in person or by eConsult. As to physician extenders, I am not convinced that they will add to the capacity without compromising the quality of care’ |
| 13. Pan-Canadian licensing for psychiatrists should be considered due to inequitable distribution of psychiatrists in Canada and advances in virtual care post COVID-19 pandemic. | ‘I am not aware of evidence that supports open licensure as a way to improve access. Rather, it may worsen it due to funding differences.’  ‘Most psychiatric care required informed knowledge of local community resources and the need to work in teams with other local professionals. Fragmenting the number of psychiatrists working in remote areas thru virtual care will not improve the accountability and the quality of team-based practice.’ |
| **Issues Discussed That Were Not Included in the Consensus Statements** | **Revised Statement** |
| 15.Although the majority of chairs supported the idea of reducing the length of general psychiatry training to 4 years from the current 5 years (similar to US training, and some other specialties), ACPC could not reach a consensus on this issue. Further work is needed to explore the similarities and differences between Canadian and US training and whether some Canadian schools may want to pilot an accelerated 4-year psychiatry residency program similar to the US. | ‘I disagree with the reducing the duration of training, as even with a 5-year training, many residents do not feel competent to practice independently. If reduction in training is a consideration, then we need to look at increasing their caseloads to ensure they get adequate experience and training’  ‘I agree but did not realize the “majority” requests this. I thought it was otherwise.’ |
